# Flash Ostial Balloon in Right Internal Mammary Artery Percutaneous Coronary Intervention: A Novel Approach

**DOI:** 10.7759/cureus.1537

**Published:** 2017-08-02

**Authors:** Rupak Desai, Gautam Kumar

**Affiliations:** 1 Research Coordinator, Atlanta Veterans Affairs Medical Center; 2 Atlanta Va Medical Center, Emory University

**Keywords:** ostial stenosis, flash ostial balloon, restenosis, right internal mammary artery, coronary artery disease

## Abstract

Despite the widespread use of coronary stents and effective anticoagulation regimens, the treatment of ostial lesions is limited by high restenosis rates. Ostial stenosis is a technically difficult condition to treat but this novel technique shows the ability to enable the optimal coverage of the ostium with excellent stent flaring using a Flash ostial balloon (Cardinal Health Inc., Dublin, OH).

## Introduction

This case illustrates the use of the Flash ostial system (Cardinal Health Inc., Dublin, OH) to achieve excellent stent apposition in a newly developed ostial stenotic lesion years after coronary artery bypass grafting (CABG). This is one of the few reported cases of Flash ostial balloon use in the right internal mammary artery (RIMA) percutaneous coronary intervention (PCI). In such cases, optimum treatment is of importance because of high restenosis rates.

## Case presentation

**Figure 1:**
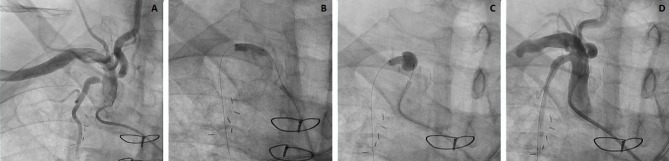
Flash Ostial Balloon in RIMA PCI *1A*- RIMA angiography revealing significant stenosis involving the ostium. *1B*- Stent deployment in RIMA ostium with intentional overhang into the right subclavian artery. *1C*- Flash ostial balloon used to flare the proximal end of the stent. *1D*- Final angiographic result showing excellent apposition of stent and re-creation of the funnel-shaped ostium. RIMA- Right internal mammary artery; PCI- Percutaneous coronary intervention

A 68-year-old male with known coronary artery disease (CAD) presented with two shocks from his implantable cardioverter-defibrillator (ICD) in the three days prior to admission. The patient had a history of CABG 20 years ago with five grafts, out of which only three were patent on angiography: left internal mammary artery (LIMA) to left anterior descending artery (LAD), reversed saphenous vein graft (SVG) to posterior descending artery (RPDA), and right internal mammary artery (RIMA) to first obtuse marginal branch (OM1). Following surgery, he developed ischemic cardiomyopathy with a left ventricular ejection fraction of 25% on optimal heart failure management. Images from the RIMA angiography are shown (Figure 1A, Video 1). A 4.5 x 12 mm bare metal stent was deployed in the RIMA (Figure 1B, Video 2) with a three mm overhang intentionally into the right subclavian artery and the ostium was flared using a 4.0 x 8.0 mm Flash ostial balloon (Figure 1C, Video 3). This yielded an excellent angiographic result (Figure 1D, Video 4). On two-year follow-up, the patient is doing very well.

**Video 1:** RIMA Angiography RIMA- Right internal mammary artery

**Video 2:** Stent Positioning in RIMA Ostium RIMA- Right internal mammary artery

**Video 3:** Flash Ostial Balloon Used to Flare the Proximal End of the Stent

**Video 4:** Final Angiographic Result

## Discussion

It is found that the long-term patency of RIMA is excellent, almost equivalent to LIMA and surely better than radial or saphenous grafts. Atheromatous changes were not found in RIMA angiograms. RIMA is, therefore, strongly recommended to be utilized in grafting [1].

In this patient, an ostial stenosis was noted several years after CABG. Ostial stenosis constitutes a therapeutic challenge for interventional cardiologists. The percutaneous treatment of coronary and saphenous vein graft aorto-ostial stenosis has been linked to lower procedural success rates, more frequent in-hospital complications, and greater chances of late restenosis compared to the treatment of nonostial lesions [2].

Stent misplacement from the true ostium has been witnessed in 54% of cases in which the right coronary artery was more commonly involved [3]. Even an experienced operator can find stenting highly challenging with this extent of geographic miss. An intravascular ultrasonographic analysis study by Castagna et al. showed stent under-expansion as the most common factor for in-stent restenosis (ISR) lesions [4].

To overcome the difficulties caused by restenosis and for a better post-procedural outcome, we present this novel approach with the Flash ostial balloon. The dual balloon design combines a higher-pressure dilatation balloon with an oversized low-pressure anchoring balloon. Due to the similarity with the natural anatomy of the funnel-shaped ostium, the Flash ostial system is used to maintain the position of the catheter at the aorto-ostial junction and to achieve excellent stent wall apposition post-dilatation in technically challenging ostial lesions. This is one of the very few reported cases of Flash ostial balloon usage in RIMA.

## Conclusions

Ostial lesions are challenging even for the experienced interventional cardiologist. Harnessing new techniques, such as the Flash ostial balloon, enables us to achieve mastery in this complex lesion subset.
